# Temporal Allocation of Foraging Effort in Female Australian Fur Seals (*Arctocephalus pusillus doriferus*)

**DOI:** 10.1371/journal.pone.0079484

**Published:** 2013-11-14

**Authors:** Andrew J. Hoskins, John P. Y. Arnould

**Affiliations:** School of Life and Environmental Sciences, Deakin University, Burwood, Victoria, Australia; Hokkaido University, Japan

## Abstract

Across an individual's life, foraging decisions will be affected by multiple intrinsic and extrinsic drivers that act at differing timescales. This study aimed to assess how female Australian fur seals allocated foraging effort and the behavioural changes used to achieve this at three temporal scales: within a day, across a foraging trip and across the final six months of the lactation period. Foraging effort peaked during daylight hours (57% of time diving) with lulls in activity just prior to and after daylight. Dive duration reduced across the day (196 s to 168 s) but this was compensated for by an increase in the vertical travel rate (1500–1600 m·h^−1^) and a reduction in postdive duration (111–90 s). This suggests physiological constraints (digestive costs) or prey availability may be limiting mean dive durations as a day progresses. During short trips (<2.9 d), effort remained steady at 55% of time diving, whereas, on long trips (>2.9 d) effort increased up to 2–3 d and then decreased. Dive duration decreased at the same rate in short and long trips, respectively, before stabilising (long trips) between 4–5 d. Suggesting that the same processes (digestive costs or prey availability) working at the daily scale may also be present across a trip. Across the lactation period, foraging effort, dive duration and vertical travel rate increased until August, before beginning to decrease. This suggests that as the nutritional demands of the suckling pup and developing foetus increase, female effort increases to accommodate this, providing insight into the potential constraints of maternal investment in this species.

## Introduction

In order to maximise reproductive success, individuals must efficiently accumulate resources to invest in reproductive endeavours. Efficient foraging (behaviours that minimise cost while maximising gain) will improve lifetime fitness and be selected for over time [1], [2]. Thus, the allocation of foraging effort should be partitioned to times where overall energetic gains are greatest [3], [4]. Conversely, other necessary behaviours, such as rest and travel, should occur at times of reduced foraging efficiency. For example, in the little brown bat (*Myotis lucifugus*) foraging duration is inversely linked to prey density with individuals spending more time resting on nights when prey densities are low [5].

At evolutionary timescales, adaptations maximising foraging efficiency will be selected for. At the ecological level (i.e. an individual's lifetime), foraging decisions will be shaped and/or constrained by multiple intrinsic and extrinsic drivers. These include the type of resource being gathered, such as, a predator being limited in foraging time by the behaviour of their prey [6]. Physiological systems and constraints will also act to shape a behavioural response. For example, reliance on internal oxygen stores limit the time air-breathing marine predators can spend in their foraging zone and, thus, animals must balance foraging behaviour with the need to replenish oxygen supplies [7], [8]. Furthermore, foraging behaviour will be shaped by life history traits of the species. For example, animals adopting a central place foraging strategy during offspring provisioning are constrained in foraging range by the duration they can leave their offspring alone [9].

Across an individual's life, the intrinsic and extrinsic factors shaping foraging behaviour may act on individuals differently at different temporal scales [10]. Thus, individual foraging decisions, and the factors driving them, may vary depending on the timescale being assessed. Timescales may work in a hierarchical structure with activities occurring at smaller scales being nested within larger scale activities. Factors driving foraging decisions at a daily scale, such as the limited capacity of an individual's digestive tract or the exposure to predation risk [11], [12], may be nested inside factors influencing foraging decisions acting at larger scales (e.g. seasonal fluctuations of resources; [13]–[15]). Thus, conclusions drawn from behavioural data will depend on the timescale being assessed and to advance understanding of a species' ecological role, data should be assessed at multiple, biologically relevant timescales. For example, once introduced to a novel environment, elk (*Cervus elaphus*) exhibit multiple movement modes, structured into several nested temporal scales [16]. Over several years, individuals shift from dispersive into home-ranging behaviour [16]. At an intermediate scale (within years), individuals alternate between minimal movement and bursts of rapid straight travel, suggesting movement between habitats [16]. Finally, at fine-scales (within days), individuals switched between movement and foraging, with foraging durations defined by the quality of the current food patch [16].

During lactation, female otariid seals (fur seals and sea lions) adopt a central place foraging strategy [17]. Individual's must return periodically to the colony to feed young and are, thus, constrained in foraging time by the fasting capabilities of their young [18]. Furthermore, as air-breathing vertebrates, otariid seals have the added complication of needing to periodically return to the surface to breathe. Individuals must allocate time efficiently to both maximise their chances of prey encounter while also ensuring they minimize the cost of breath-hold diving [19]. Thus, foraging decisions must occur at multiple temporal scales. At the smallest scale individuals must make decisions within a single dive cycle which will be driven by the prey encountered during the dive, or on previous dives, and the individual's current aerobic capabilities [20]. Dives may be nested within bouts of dives whose duration may be affected by the size of the prey patch, individual digestive constraints and predator avoidance tactics [21]–[24]. Foraging bouts, and their timing, may then be nested into single days where individuals might be influenced by their own or their prey's circadian rhythms [25]. These are further nested within multiple day foraging trips, where individuals alter behaviour in response to prey densities and whose duration is limited by the fasting capabilities of their young [26], [27]. Foraging effort/decisions may also be influenced by seasonal variations in prey availability or nutritional demands of pups with stage of lactation [13], [28], [29].

Temporal variations in foraging effort have been shown at different scales in several otariid seal species [24], [28]–[32]. Pelagic foraging species are known to optimize diving effort during times when prey capture is most efficient (e.g. nocturnal diving by species targeting vertically migrating prey; [28], [30], [31]) while also increasing foraging effort seasonally with the increasing demands of their young [32]. In contrast, in benthically foraging species, temporal variations in foraging effort have been less readily identified and it has been suggested this may be a common feature for species occupying this niche [24].

In the present study, the temporal structure of foraging effort in the Australian fur seal (*Arctocephalus pusillus doriferus*, hereafter referred to as AUFS), a benthically foraging species that is endemic to the shallow continental shelf region of Bass Strait in south-eastern Australia, was investigated [33]–[36]. Previous studies of the diving behaviour of this species have found limited temporal structure in their foraging behaviour, with no bout structure and only a slight increase in daytime foraging observed during the winter for females [36]. There is little information, however, on how the species allocates foraging effort at different time scales. This information is important for understanding the drivers of foraging success, the limits of behavioural adaptations within the species and how these may be influenced by environmental variability.

The aims of the present study, therefore, were to detail how female Australian fur seals temporally allocate foraging effort, and some of the different diving behaviours that define it, within three different time scales that are considered to be influential to foraging decisions made by this species: 1) diel variation (assessing the potential influence of daily patterns of prey availability); 2) between days at the scale of the foraging trip (assessing potential foraging decisions being made across multiple days/prey patches while limited by the needs of their fasting young); and 3) across foraging trips during the final six months of lactation (assessing the potential influence of the period of greatest nutritional demand of the nursing young and developing foetus).

## Methods

### Ethics statement

All work was carried out with approval of the Deakin University Animal Ethics committee and under Department of Sustainability and Environment (Victoria, Australia) wildlife Research Permits (10000187, 10000706, 10001143, 10001672, 10005362, and 10005848). Kanowna Island is part of the Wilsons Promontory Marine National Park and was accessed under permit from Parks Victoria.

### Study site and animal handling

The study was conducted between late-March and September of 1998–2009 on Kanowna Island (39° 9.1′S, 146° 18.5′E), northern Bass Strait, south-eastern Australia. Lactation for this species extends from the birth of the pup between November and December (peak late-Nov to early-Dec), lasting approximately 10 months [37], [38]. As such, female behaviour was assessed while pups were aged between 4 and 10 months old. Kanowna Island hosts a large colony of AUFS with an annual pup production of approximately 3400 pups, representing 13.3% of the total population of this species [39]. Individuals from this colony have been shown to focus foraging to the south-west of the colony in the central Bass Strait basin, where there is minimal variation in the bathymetric profile (averaging depth of 86 m; [33], [34]).

Lactating females nursing pups were selected at random and captured using a modified hoop net (Fuhrman Diversified, Seabrook, Texas., U.S.A.), manually restrained and administered an intramuscular injection of Midazolam (0.1 mg·kg^−1^, Hypnovel®; Roche Products Pty. Ltd., Dee Why, New South Whales, Australia) into the gluteus muscle. Prior to 2002, procedures were conducted solely while the animal was sedated and held in a restraint board [33], [36], [40]. Thereafter, individuals were anaesthetised using isoflurane delivered via a portable gas vaporizer (Stinger™, Advanced Anaesthesia Specialists, Gladesville, NSW, Australia.; [41]) following induction with Midazolam.

A dive behaviour recorder sampling at 5 or 1 s intervals (Mk07, Mk08, Mk09, Mk10; Wildlife Computers, Redmond, WA, U.S.A.) and a VHF transmitter (Sirtrack Ltd, Havelock North, New Zealand) were then glued in series along the midline of the dorsal pelage, just posterior to the scapula, using a quick-setting epoxy (Accumix 268, Huntsman Advanced Materials Pty Ltd, Deer Park, Vic, Australia). To aid in identification, individually-numbered plastic flipper tags (Super Tags®, Dalton Supplies, Woolgoolga, NSW, 2456, Australia) were then inserted into the trailing edge of each fore-flipper. Upon recovery from sedation/anaesthesia, individuals were released and left to resume normal behaviours. After one or more foraging trips to sea, the animals were recaptured using the same method described above and devices were removed by cutting the fur beneath. Individuals were then released back into the colony.

A number of individuals (40%) in the study were also instrumented with a FastLoc™ GPS transmitter (FastLoc™ 1, Sirtrack Ltd.), satellite transmitter (KiwiSat100, Sirtrack Ltd) or heart-rate logger (HTR, Wildlife Computers Ltd.) for use in concurrent studies. Devices were glued in series with dive behaviour recorders and VHF transmitters to minimise additional drag and, in all cases, total instrument packages weighed <2% of body mass and represented <1% of cross sectional surface area. These additional devices would have had minimal extra impact on the diving behaviour of the animals in the current study [42]. Data recorded by these devices were not included in the current study.

### Data analyses

Data were downloaded from the dive behaviour recorders onto a portable computer in the field before being prepared for re-deployment. Downloaded data were extracted from the Wildlife Computers proprietary format using Instrument Helper (Wildlife Computers.). Drift in the pressure readings of devices (zero offset errors) were corrected and subsequent summary dive statistics (time of dive, dive duration, maximum depth and post-dive interval duration) were calculated using the diveMove package [43] within the R statistical environment [44]. A foraging trip was defined as continuous periods >6 h spent in the water within which at least one dive occurred. This time was chosen as Australian fur seals have been observed to spend several hours at a time in the water surrounding the colony for purposes other than foraging, such as thermoregulation (*personal observation*). Haul-out periods were defined as when the animal was out of water >10 min.

Summary dive statistics were aggregated into hourly blocks and, for each hourly block, one index of foraging effort (i.e. at-sea energy expenditure) and three indices of diving behaviour were calculated. The proportion of time spent diving has been shown to be a good indicator of at-sea energy expenditure in otariids [45] and was used in the current study to give an overall index of foraging effort. This index is the cumulative result of three other diving metrics: the number of dives within the time period, the duration of each dive, and the interval between dives. To allow for a fine-scale analysis of the use of foraging effort, these were represented in the current study by calculating three other metrics: the vertical travel rate (the total vertical distance, ascent and descent, travelled during a time period. unit: m·h^−1^); mean dive duration (s); and mean postdive duration (s).

Using a regression modelling approach (see details below), temporal variation in these indices was then investigated at three scales: daily (hour of the day); intra-trip (number of days into a foraging trip); and inter-trip (Julian day) variation. Model assumptions were first checked by assessing the distribution of each response variable through the use of histograms, qq plots, boxplots and empirical cumulative distribution plots [46], [47]. Colinearity between predictors was then assessed by calculating variance inflation factors and correlation coefficients using the AED package (AED ver 1.0) in the R statistical environment. Finally, scatter plots were produced of predictor and response variables to determine the nature of the response being modelled. Based on these exploratory analyses, and the nested nature of the data (i.e. multiple individuals each containing multiple trips), data were modelled using Generalised Additive Mixed effects Modelling (GAMM). Autocorrelation was accounted for using an autoregressive correlation structure of order one (AR1), while within group heteroscedasticity was corrected using either a power or exponential variance function. Models were run using the R package mgcv (mgcv ver 1.7-22). Smooth terms were fit to all predictor variables using penalized thin plate regression splines (number of days into foraging trip and Julian day) and cyclic cubic regression splines (hour of the day). Gaussian distributions with identity link functions were used for all response variables. Repeated measures and individual variation were accounted for in the model by setting the year of deployment, individual seal and individual foraging trip as random effects.

Individuals may also alter foraging effort by altering the length of time they spend away from the colony foraging or by altering the duration of the time they spend on land between foraging trips to increase time spent foraging overall. To assess the temporal relationships between foraging trip duration, haul-out duration and foraging effort, a regression modelling approach was adopted. Firstly, a Linear Mixed Effects model (LME) was developed to look the response of foraging trip duration (h) to the predictor variables foraging effort (proportion of time diving within a foraging trip) and to assess temporal trends in foraging trip duration month of the year was included as a categorical fixed factor. Also included within this model was the interaction term between foraging trip duration and month of the year. Secondly, the response of total foraging effort (proportion of time spent foraging during a foraging cycle, i.e. foraging trip duration + haul-out duration) was modelled against month of the year in a mixed effect ANOVA. Finally, the response of haul-out (h) duration to foraging trip duration and month of the year, as well as the interaction between the two, was assessed using another LME.

When determining what month a foraging trip belonged to, if a foraging trip straddled two months, the month that contained the greatest proportion of the trip was used. Prior to modelling, model assumptions were checked using methods described above and, to provide sufficient numbers within each random effect grouping, the data were further reduced to only contain records from individuals containing three or more foraging trips. Repeated measures and individual variation was accounted for by using year of deployment and individual seal as random effects within the models. Temporal autocorrelation and between group heteroskedascity were accounted for using an AR1 correlation structure and power variance function within all models. Model selection occurred though a backwards-stepwise selection procedure whereby the least significant term (selected by Akiake's Information Criterion; AIC) in the model was dropped and the resultant model was re-run until all terms were significant.

Unless otherwise stated, all analyses were carried out following the methods of Zuur *et al* [47], Wood [46], Pinheiro and Bates [48]. Data are presented as mean ± standard deviation when assumptions of normality are met or as the mode when they are not.

## Results

Complete dive behaviour records were obtained from a total of 68 individuals, covering a total of 547 foraging trips. Deployment durations ranged 3.9–142.4 d (31.5±35.0 d) covering an average of 8.2±8.8 foraging trips per individual (Table S1). Individual foraging trips lasted for an average of 2.9±2.4 d (range 0.5–13 d). During these foraging trips, individuals made 201.5±62.5 dives per day, averaging 186.9±54.4 s in duration with an average post-dive duration of 264±410 s. On average, individuals had a diving rate (vertical travel rate) of 1133±545 m·h^−1^ and a modal dive depth of 79.8±23.5 m. This corresponds to individuals spending on average 40.0±0.1% of each day at sea submerged in foraging dives (Table S1). Dive depths were extremely consistent with 70% of dives occurring within 6.2 m of the modal depth achieved by an individual. The distribution of maximum depths achieved also tended to have a strong negative skew (mean skewness: −2.2±2.1), indicating that individuals tended to dive at or close to the seafloor for the majority of their dives.

### Temporal variations in foraging effort

The GAMM results indicated the four indices of foraging effort (proportion of time spent diving, mean dive time, mean postdive duration and vertical travel rate) varied significantly at the three temporal scales investigated. To provide a clear description of the structure of diving behaviour at each of the time scales assessed, the results are presented grouped by the predictor variables (hour of the day, number of days into the foraging trip and Julian day) rather than grouping the results by individual models (Table 1).

**Table 1:** Summary results of the four Generalised Additive Mixed Effects Models used to assess the temporal trends in the foraging effort of female Australian fur seals provisioning young at Kanowna Island, Bass Strait, Australia.

Response variables	Predictor variables	Parametric coefficients			Approximate significance of smooth terms	
		Est	SE	*t*	edf	*F*
Proportion of time diving	intercept	0.51	0.01	39.49		
	hour of day				10.69	46.35
	Julian day				5.27	16.77
	n days into trip				6.10	141.81
Mean dive duration (s)	intercept	184.10	3.87	47.57		
	hour of day				9.15	51.80
	Julian day				4.72	58.79
	n days into trip				9.17	23.88
Vertical dive effort (m/hr)	intercept	1303	36.26	35.94		
	hour of the day				9.85	192.18
	Julian day				6.10	53.27
	n days into trip				7.9	11.56
Mean postdive duration (s)	intercept	105.50	2.36	44.65		
	hour of the day				13.05	12.37
	Julian day				6.318	28.57
	n days into trip				1.74	33.97

Est: estimated parametric coefficient. SE: estimated standard error of parametric coefficient. Non-parametric smooth terms from these models have been, later, presented grouped by the predictor variables (each timescale), rather than the individual model.

All *p*-values were <0.0001.

**Figure 1:**
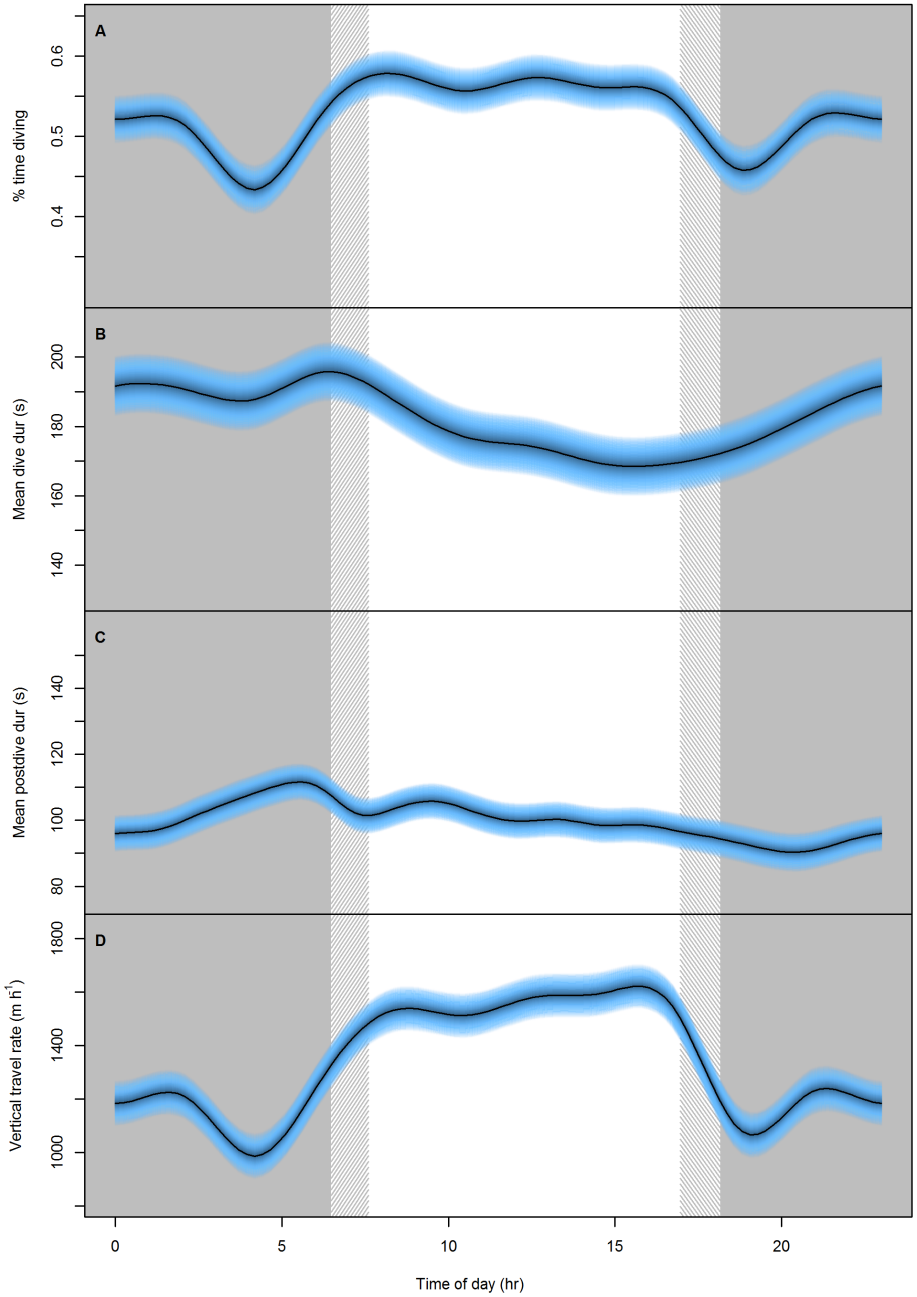
Foraging effort response to time of day. The predicted response of A) proportion of time diving (%), B) mean dive duration (s), C) mean post-dive duration (s) and D) vertical distance covered (m·h^−1^) in female Australian fur seals to different times of the day. Grey areas represent local night time and the hatched regions represent the band of time that sunrise/sunset occurred over the study period. Blue bands represent the 95% confidence intervals around the predicted response. (see Table 1 for statistical results).

The proportion of time spent submerged varied in a curvilinear fashion during the course of the day with individuals displaying the greatest proportion of time diving (0.57–0.56) between 08:00 and 15:00 local time (Figure 1 A). Prior to, and after, this daytime peak, there were distinctive lulls in diving activity (Figure 1 A). A second, less pronounced peak of diving activity was evident between 21:30 and 01:30. Similarly, mean dive duration peaked in the morning at approximately 06:00 (196 s) with a second peak during the night just after midnight (192 s) (Figure 1 B). However after the morning peak, mean dive duration steadily declined throughout the day until approximately 15:00 to 168 s before increasing again during the night (Figure 1 B). Correspondingly, post-dive duration also decreased throughout the day from a peak just after 05:00 (111 s), indicating individuals were making shorter, more frequent dives as the day progressed (Figure. 1 C). This is consistent with vertical travel rate (m·h^−1^) being highest, and increasing slightly, during the daylight hours (Figure 1 D). However, minimum post-dive duration (90 s) was not reached until well after sunset at approximately 20:00–20:30, at a time when dive duration was increasing.

**Figure 2:**
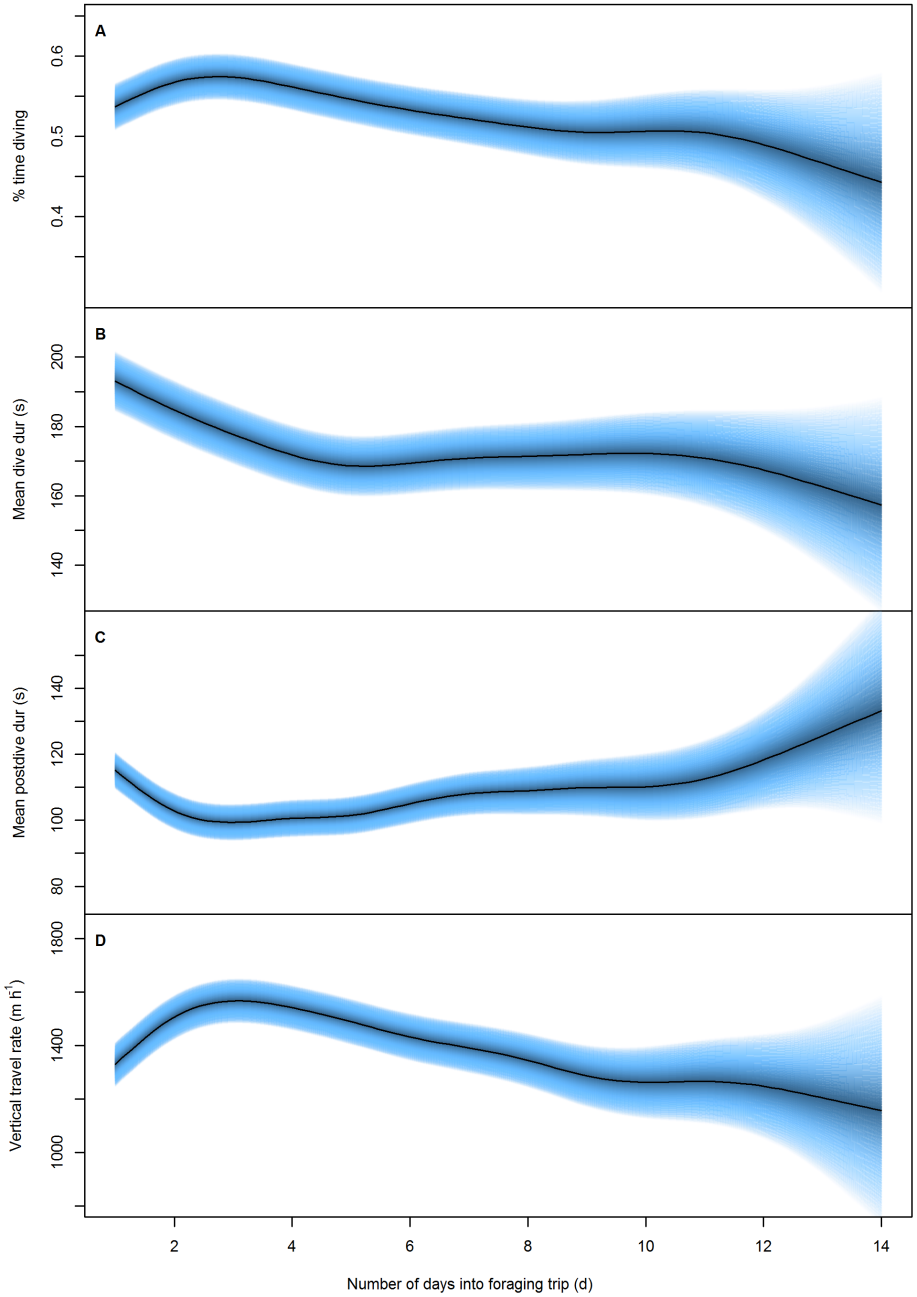
Foraging effort response to number of days into foraging trip. The predicted response of A) proportion of time diving (%), B) mean dive duration (s), C) mean post-dive duration (s) and D) vertical distance covered (m·h^−1^) in female Australian fur seals to the number of days into a foraging trip. Blue bands represent the 95% confidence intervals around the predicted response. (see Table 1 for statistical results).

Throughout the course of foraging trips, the proportion of time spent diving increased for the first 2–3 days to 57% before decreasing steadily for the remainder of the time the animal was at sea (Figure 2 A). Dive duration decreased rapidly from 192 s over the first five days of foraging trips before levelling at approximately 168 s for the remainder of the time at sea (Figure 2 B). Similarly, mean post-dive duration decreased from 114 s during the first 2–3 days before starting to gradually increase for the remainder of the time at sea (Figure 2 C). Correspondingly, the vertical travel rate increased from 1328 m·h^−1^ at the beginning of the trip to a peak of 1564 m·h^−1^ at day 3 before gradually decreasing in trips that lasted greater than three days (Figure 2 D).

**Figure 3:**
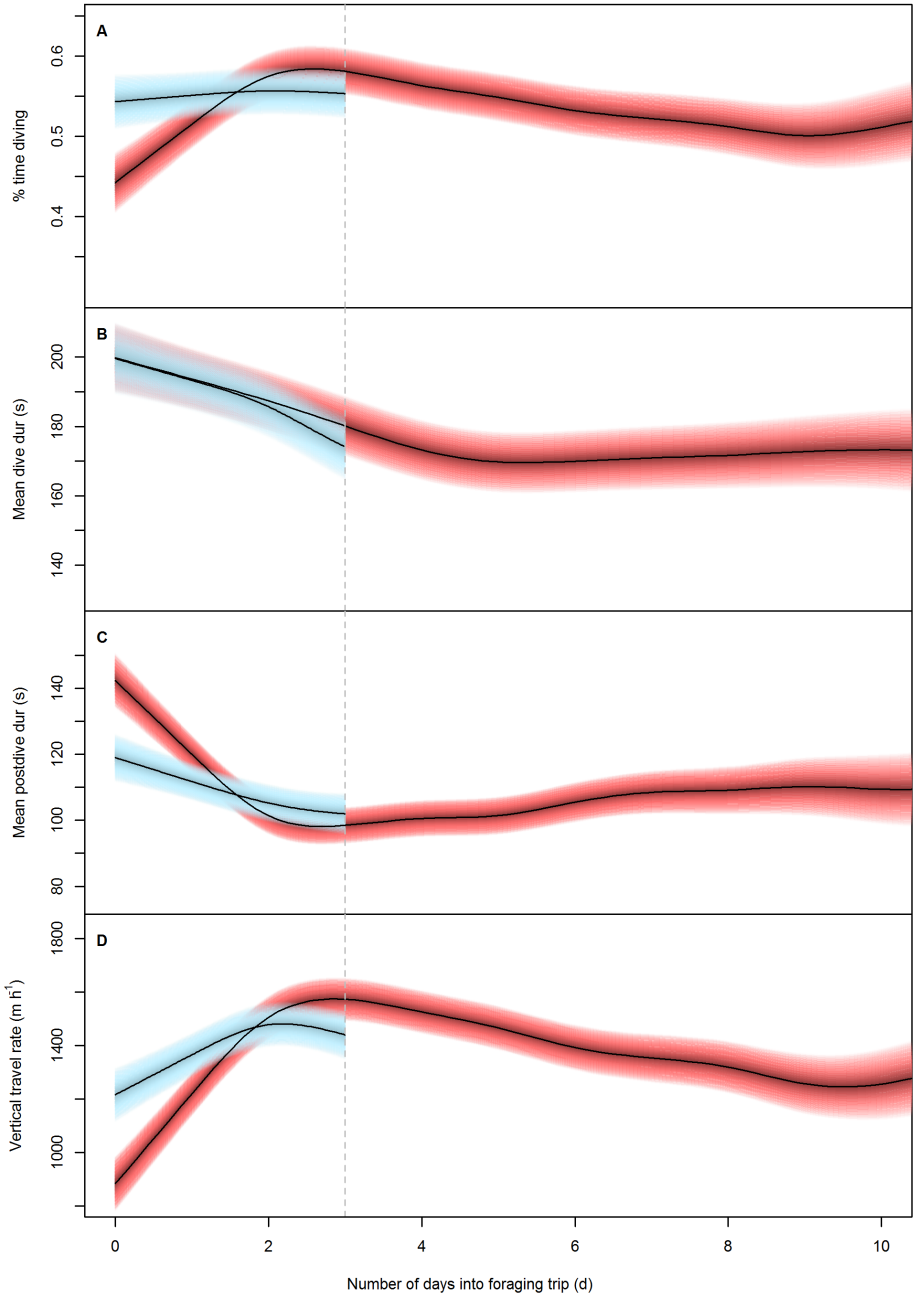
Foraging effort response to number of days into short and long foraging trips. The predicted response of A) proportion of time diving (%), B) mean dive duration (s), C) mean post-dive duration (s) and D) vertical distance covered (m·h^−1^) during short (light blue) and long (red) foraging trips in female Australian fur seals to the number of days into a foraging trip. Blue bands represent the 95% confidence intervals around the predicted response. (see Table 2 for statistical results). Note: limits of x-axes have been restricted to 10 days to improve distinction between the responses of short and long foraging trips (i.e. the first three days of long trips). For more information on the predicted responses of long foraging trips exceeding 10 day refer to figure 2.

The peak in foraging effort found in the intra-trip part of the analysis (Figure 2 A) corresponded closely with the mean foraging trip duration of the dataset (2.9±2.4 d). Thus, a significant proportion of individual foraging trip durations were at or below the peak identified in Figure 2 A, indicating the potential for a different response of foraging effort for individuals undergoing short compared to long foraging trips. To assess this, models were rerun with separate smoothing splines fit in the number of days into a foraging trip for individuals undergoing short (<2.9 days, n = 45 individuals and 288 foraging trips) and long (>2.9 days, n = 59 individuals and 204 foraging trips) foraging trips. Results of these models showed that there was a difference in the foraging effort response between individuals undergoing short and long foraging trips (Table 2). During short foraging trips, individuals maintained a consistent high proportion of time spent diving (55%) whereas individuals undergoing long trips gradually increased the proportion of time diving until a peak at 2–3 days (44%–58%; Figure 3 A). However, mean dive duration showed an almost identical decline between short and long foraging trips with the only difference being a slight increase in the gradient of decline at the end of a short foraging trip (Figure 3 B). Interestingly, the minima reached at the end of short foraging trips (174 s) is near to the minima reached at approximately five days (168 s) while individuals are undergoing long foraging trips. The mean postdive duration and vertical travel rate showed similar shaped responses during the first 2.9 days of a foraging trip (Figure 3 C and D). However when undergoing long foraging trips, individuals showed a much more pronounced response, with much higher/lower starting postdive durations and vertical travel rate that decreased/increased at a much steeper rate when compared with short foraging trips (Figure 3 C and D).

**Table 2:** Summary results of the four Generalised Additive Mixed Effects Models, the include individual smoothers for short and long foraging trips, used to assess the temporal trends in the foraging effort of female Australian fur seals provisioning young at Kanowna Island, Bass Strait, Australia.

Response variables	Predictor variables	Parametric coefficients			Approximate significance of smooth terms		*P*
		Est	SE	*t*	edf	*F*	
Proportion of time diving	Long trip	0.51	0.01	39.53			<0.0001
	Short trip	−0.02	0.01	−1.56			0.118
	hour of day				9.65	57.42	<0.0001
	Julian day				8.04	14.96	<0.0001
	n days: short trips				1.81	0.30	0.714
	n days: long trip				6.68	23.55	<0.0001
Mean dive duration (s)	Long trip	184.83	3.91	47.27			<0.0001
	Short trip	−11.69	3.42	−3.41			0.0006
	hour of day				7.764	72.53	<0.0001
	Julian day				9.18	20.91	<0.0001
	n days: short trips				1.681	15.05	<0.0001
	n days: long trip				4.60	52.17	<0.0001
Vertical dive effort (m/hr)	Long trip	1288	34.39	37.46			<0.0001
	Short trip	−79.76	43.81	−1.82			0.068
	hour of the day				9.82	206.02	<0.0001
	Julian day				8.39	11.42	<0.0001
	n days: short trips				1.94	13.82	<0.0001
	n days: long trip				7.09	66.23	<0.0001
Mean postdive duration (s)	Long trip	106.26	2.40	44.24			<0.0001
	Short trip	−6.19	2.16	−2.86			0.004
	hour of the day				11.44	16.93	<0.0001
	Julian day				1.66	37.45	<0.0001
	n days: short trips				2.19	12.81	<0.0001
	n days: long trip				6.77	28.56	<0.0001

Est: estimated parametric coefficient. SE: estimated standard error of parametric coefficient.

Note: Only results of the number of days into the foraging trip part of the analysis shown as the shape of response for other timescales did not vary significantly from those presented using models without individual smoothing splines for short and long foraging trips.

**Figure 4:**
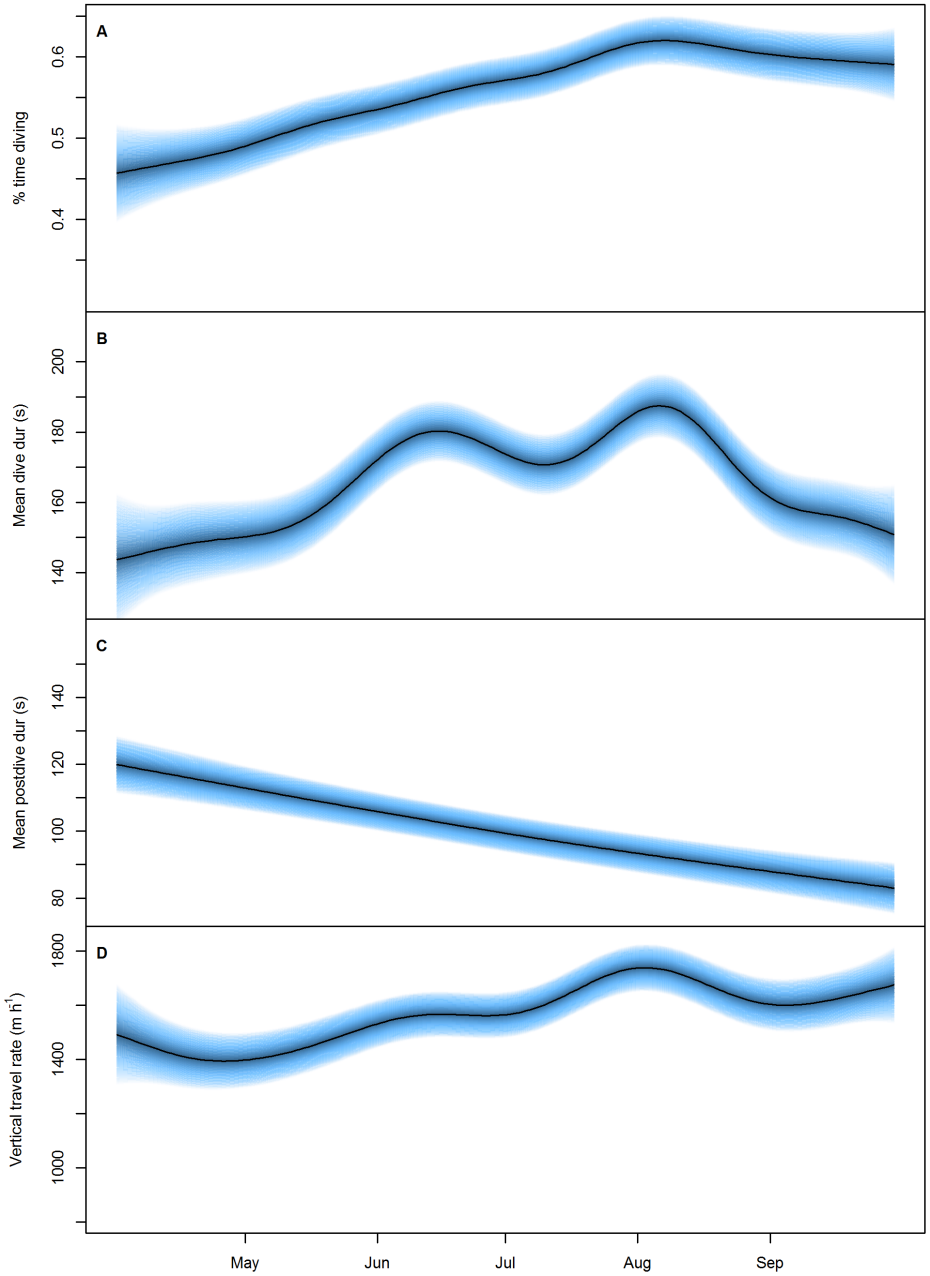
Foraging effort response to time of year. The predicted response of A) proportion of time diving (%), B) mean dive duration (s), C) mean post-dive duration (s) and D) vertical distance covered (m·h^−1^) in female Australian fur seals to the time of year. Proportion of time diving calculated from the period individuals were at-sea and does not include haul-out periods. Blue bands represent the 95% confidence intervals around the predicted response. see Table 3 for statistical results).

During the inter-trip variation period of investigation (April to September), the proportion of time spent diving increased from 45% to a peak of 61% in mid-August before decreasing slightly (Figure 4 A). Correspondingly, dive duration increased from a mean of 142 s in April, displaying two peaks at 180 s and 187 s in June and August, respectively, before decreasing for the remainder of the study period (Figure 4 B). Post-dive duration, however, decreased consistently from 119 s to 82 s throughout the study period (Figure 4 C). Vertical travel rate generally increased through the study period peaking in August (1732 m·h^−1^) at the same time as dive duration and the proportion of time spent diving peaked (Figure 4 D). These indices suggest that females provisioning pups increase their foraging effort throughout the winter months by conducting long dives with short inter-dive intervals whereas in spring they continued to maintain a high foraging effort by conducting shorter dives more frequently.

**Figure 5:**
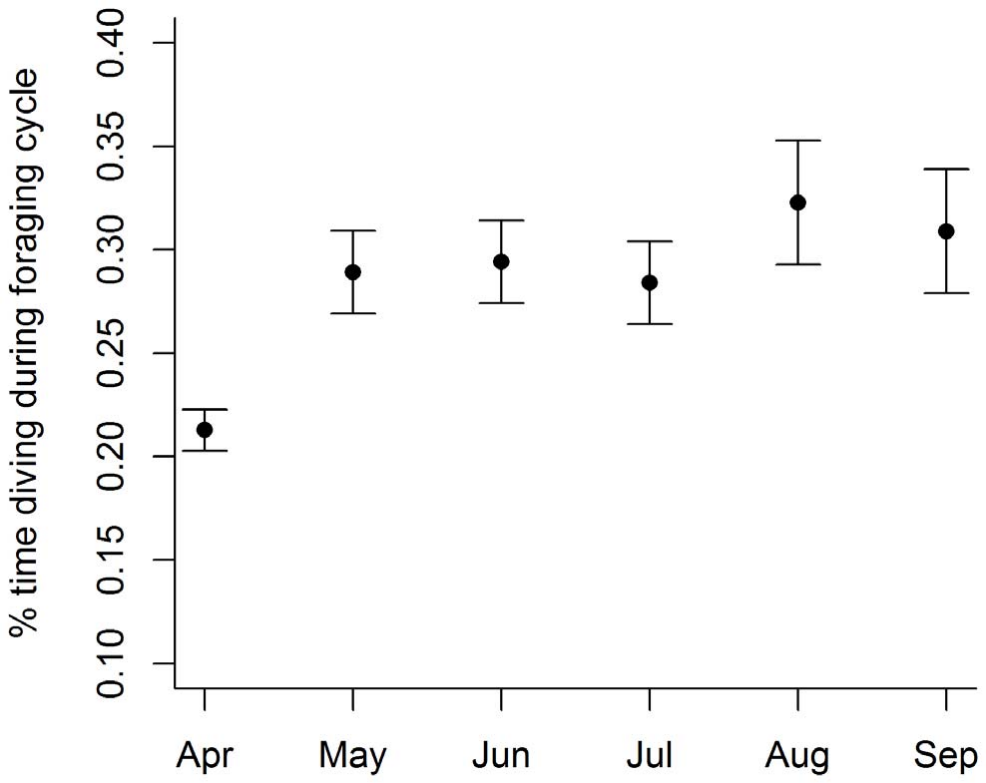
Seasonal effects on foraging effort within foraging cycle. Differences in the proportion of time spent diving during the whole foraging cycle (foraging trip duration+haul-out duration) throughout the second half of the lactation period for female Australian fur seals provisioning young at Kanowna Island, Bass Strait, Australia. (values taken from the coefficient estimates and corresponding standard errors of the LME model. see Table 3 for statistical results).

A significant negative relationship was found between foraging trip duration and the proportion of time spent diving during the foraging trip (Table 3). However, no relationship was found between month of the year and foraging trip duration (Table 3). Furthermore, the mixed effects ANOVA assessing the relationship between total foraging effort (proportion of time spent diving during the foraging cycle) found total foraging effort to vary significantly between months (Table 3). Foraging effort in April was lowest for the study period (prop time diving: 0.21±0.01) with the general tread of foraging effort increasing (with a plateau between May–July) throughout the study period (Figure 5). These results suggest that although overall foraging effort increases throughout the study period, it is not a result of increasing foraging trip durations; rather AUFS achieve this by increasing their diving effort within a foraging trip.

**Table 3:** Summary results of models assessing the response of foraging trip duration (h) to the corresponding proportion of time spent diving and time of year, the response of the proportion of time spent diving during a foraging cycle to the time of year and the response of haul-out duration (h) to the corresponding foraging trip duration and time of year in female Australian fur seals provisioning young at Kanowna Island, Bass Strait, Australia.

Response variable	Predictor variables	Est	SE	df_1_	df_2_	*F*	*p*
Trip duration	Intercept	113.2	16.8	1	140	71.1	<0.0001
	Prop time diving	−67.7	27.6	1	140	6.0	0.01
	Month of the year			5	140	1.4	0.20
Prop time diving (foraging cycle)	Intercept	0.21	0.01	1	141	639.4	<0.0001
	Month of the year			5	141	4.6	<0.0001
Haul-out duration	Intercept	32.4	6.3	1	257	525.6	<0.0001
	Trip Duration	0.17	0.02	1	257	116.7	<0.0001
	Month of the year			7	257	3.3	0.02

Est: estimated parametric coefficient. SE: estimated standard error of parametric coefficient.

### Factors influencing haul-out durations

**Figure 6:**
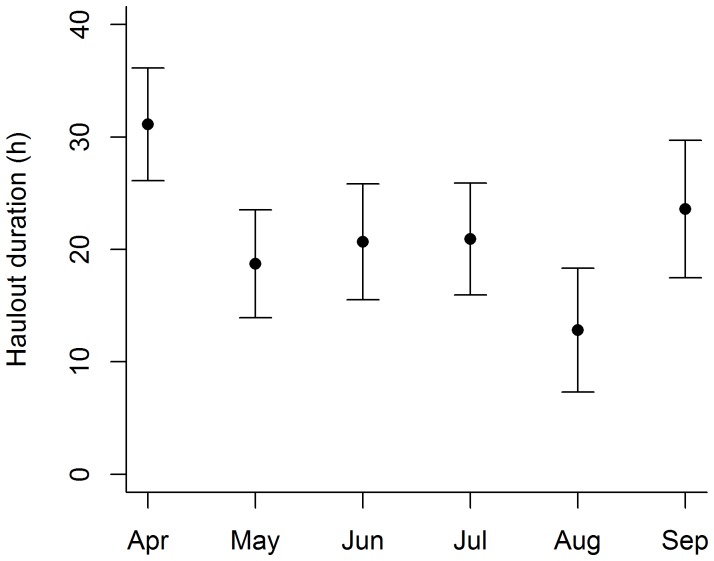
Seasonal effects on haul-out durations. Differences in the duration of haul-out periods throughout the second half of the lactation period for female Australian fur seals provisioning young at Kanowna Island, Bass Strait, Australia. (values taken from the coefficient estimates and corresponding standard errors of the LME model. see Table 3 for statistical results).

Haul-out durations lasted for an average of 31.9±23.6 h (range 0.2–155.7 h). The full LME model contained an interaction term between the month of the year and haul-out duration. However, this interaction term was removed during model selection, leaving only trip duration and month of the year as significant predictor variables within the most parsimonious model (Table 3). In the final model, trip length was able to predict haul-out duration (n = 48 animals), and month of the year significantly changed the relationship (Table 3). Haul-out duration increased on average 12 mins (0.2 h) for every additional hour a seal spent at sea (Table 3). Haul-out duration dropped from approximately 32 h in March–April to 17.1–20.8 between May–August before increasing again through September (Figure 6).

## Discussion

How animals partition foraging effort can have important implications for their immediate foraging success and long-term fitness [49], [50]. The current study found that foraging effort (proportion of time spent diving) and diving behaviour (mean dive duration, vertical travel rate and mean postdive duration) in female AUFS provisioning pups varied at multiple temporal scales. These findings provide insights into potential constraints on maternal investment in this species and the behavioural tactics used by AUFS to maintain increased foraging effort.

### Intra-trip variations in foraging effort

Within a foraging trip, central placed foragers should partition effort to the period when success is most likely and/or the least costly in terms of the energy gained per energy expended [51], [52]. For example, pelagic foraging otariid seals will focus diving during the night when their prey are nearer to the surface and easier to reach [28], [30], [53], [54]. In contrast, benthic foraging otariid seals do not generally display any diel patterns in foraging [55], [56]. Studies have suggested this is due to the availability of cryptic benthic prey not changing throughout the day [55], [56].

In the current study, foraging effort was similar throughout the day being only slightly higher during daylight hours when compared to nocturnal foraging (peak daytime effort 57%, peak night time effort 52%), consistent with what has been found previously in Australian fur seals [36]. However, the current study also detected distinct lulls in effort just prior to and just after daylight hours. These lulls in effort may represent periods where animals are resting due to decreased prey availability. Indeed, as the times correlate with the transition between light and dark (and *vice versa*) it is possible that during these times active prey go into hiding while others are yet to emerge [57], [58]. For example, jack mackerel (*Trachurus declivis*), a major prey item of AUFS, tend to school near the surface during the dawn/dusk periods and then spend the day in deeper water at or near the sea-floor, where they become most accessible to AUFS [59], [60]. Conversely, individuals may be catching larger prey during these periods with an increased level of satiation resulting in decreased diving effort [61].

Foraging effort at night was the due to increased dive durations and lower postdive durations. Many pelagic foraging species have shown increased foraging effort during the night, with individuals following vertically migrating prey [25], [28], [62]. However, AUFS forage benthically and, therefore, would not be following the vertical movements of prey. Several prey of AUFS are active nocturnally (e.g. Gurnard *Triglidae spp.*) and it is likely AUFS are targeting these species during this period [63], [64].

During the day, dive duration peaked soon after sunrise and then decreased throughout the rest of daylight hours. The concurrent decrease in post-dive duration was matched with increasing dive rate indicating individuals made more frequent, shorter dives throughout daylight hours. Australian fur seals from Kanowna Island forage primarily in the central Bass Strait basin, an area with an extremely uniform bathymetric profile [33], [34]. This results in consistent benthic dive depths (average modal depth 79.8 m with 70% of dives occurring within 6.2 m of the mode) and, thus, a reduction in dive duration would represent a decrease in foraging time on the sea floor rather than a change in maximum dive depth. This reduction of time at the sea floor is counter-intuitive in the context of optimal diving models that suggest an individual (particularly in benthic foraging species) should be maximizing its time at the bottom portion of the dive [19], [65]. The observed pattern, therefore, of decreasing dive and post-dive duration while maintaining/increasing dive rate could reflect either a change in prey availability [20], [66] and/or physiological capacity [67], [68] throughout daylight hours.

Although daily variations in prey availability have been shown in pelagic foraging pinnipeds [69], this has not been established in benthically foraging otariid seals. However, temporal patterns in prey size and feeding success have been shown in grey seals (*Halichoerus grypus*) [61]. Grey seals capture their largest prey at dawn and smallest prey during the night, with the least number of feeding events occurring during dawn [61]. Australian fur seals forage on a variety of different prey that are active at different times of the day [70], [71] and likely require different chase/capture techniques. Consequently, like grey seals, the daily variations in diving behaviour seen in the current study may reflect different prey or different sized prey being targeted or becoming available at different times of the day.

The reduction in mean dive duration observed in the current study might be evidence of the increased metabolic costs associated with digestion and assimilation of prey (i.e. Specific Dynamic Action; SDA. [72]). Rosen *et al* [22] suggested that the increase in metabolism required for digestion (SDA) is incompatible with the maintenance of maximal aerobic dive durations. Thus, to continue digesting food resources some oxygen must be diverted into digestive processes, reducing the aerobic dive capacity of an individual, or digestion must be deferred to times outside of the foraging bout [68].

In Weddell seals (*Leptonychotes weddelli*), successful foraging dives cause an increase in metabolic rate of 44.7% with metabolism remaining elevated for up to five hours post-feeding [73]. In captivity, SDA has been measured in three species of pinniped, with metabolism remaining elevated for periods of 6–8 h (small meal) and 8–10 h (large meal) in Steller sea lions (*Eumetopias jubatus*), 15 h in harbour seals (*Phoca vitulina*) and at least 10 h in harp seals (*Phoca groenlandica*) [74]–[76]. Furthermore, grey seals will delay the SDA response to periods outside of a foraging bout when foraging in deep water, however when feeding occurs in shallow water, SDA occurs accompanied by a reduction in dive durations [67], [68]. These studies suggest that SDA can affect the metabolic rate and, thus, aerobic dive capabilities of seals for extended periods of time both within and outside of a foraging bout. Therefore, it is possible that the daily patterns of diving effort observed in AUFS may be the result of increased metabolic costs associated with the digestion and assimilation of prey.

Prior to splitting foraging trips into short or long duration, there was an increase in the proportion of time spent diving to a peak at 2–3 days. However, allocation of foraging effort varied between short (≤3 d) and long (>3 d) foraging trips. During long trips, effort began low and steadily increased to peak at 2–3 days. This gradual increase in effort during the first 2–3 days of a long trip may reflect individuals primarily commuting to profitable foraging areas and increasing the level of diving effort along the way. Similarly, the reduction in effort beyond 2–3 days could be indicative of individuals beginning to leave the profitable foraging ground and return to the colony. Like other benthic foraging species [24], AUFS commence diving soon after leaving the colony and dive to the benthos while commuting rather than swim at the surface [36]. This may reflect a strategy for opportunistically encountering prey from the seafloor while travelling rather than travelling at the surface where prey would not be found and individuals may be vulnerable to predation [36]. Contrastingly, during short trips foraging effort remained consistently high for the duration of the foraging trip. The differences in effort between short and long trips suggest individuals leave the colony with some prior expectation of the duration of their foraging trip and allocate foraging effort accordingly (i.e. travel to foraging grounds and opportunistically feed along the way or begin foraging immediately with a high amount of effort).

Surprisingly, mean dive duration followed a very similar declining trajectory during the overlapping 3 days of short and long foraging trips, suggesting a similar process is acting on dive duration during short and long trips. Individuals may be transiting and/or feeding in similar habitats during these overlapping days and, thus, adopting similar foraging strategies. However, when returning from foraging trips individuals should then return through the same or similar habitat as the outward journey. If dive durations were linked to foraging habitat/strategies then individuals should show similar dive durations at the beginning and end of foraging trips. This response was not seen in either short or long foraging trips in the current study where dive duration declined before, in long foraging trips, stabilising at approximately 4–5 days.

In the Indian python (*Python molurus*) and Chinese striped-necked turtle (*Ocadia sinensis*) repetitive feeding at frequencies shorter than the time required for metabolic rate to return to base levels, results in an additive effect on the degree and duration of SDA [77], [78]. Although care should be taken when comparing between the physiological systems of such distant species, it is not unreasonable to conclude the possibility of similar additive effects on the SDA of AUFS. If the effects of SDA on metabolism were additive in AUFS, then without an extended period where feeding did not occur, the cumulative metabolic costs of repetitive feeding could show the pattern of reduced dive durations seen. Rosen and Trites [74] showed SDA from a single large feeding to last for up to 10 hours in Steller sea lions. In the current study, 75% of foraging trips had a maximum recorded postdive duration of less than 10 hours, suggesting AUFS might not always rest for sufficient time during foraging trips for metabolic rate to return to base levels

Positive relationships between foraging trip duration and the duration of subsequent haul-out periods have been found in several other species of otariid seals [79], [80]. Individuals in the current study showed the same trend and, as has been suggested in other studies, this relationship is likely due to the increased needs of their fasting young [79], [80].

### Seasonal/annual variations in effort

Australian fur seals have a synchronous breeding cycle where pups are born Nov to Dec (peak late-Nov to early-Dec) and weaned 10 months later in Sep–Oct [37], [38]. As such, the females assessed within this study would have all been nursing similar aged pups. As income breeders, AUFS must adapt their foraging effort to the increasing nutritional demands of their growing offspring [81]. As time progresses into a breeding cycle and total parental investment into the young becomes greater, the fitness benefits of providing for the offspring may outweigh personal energetic needs [82], [83]. In such situations, individuals may continue to increase foraging effort while providing resources to the young even at the cost of personal condition [82], [83]. In otariid seals, seasonal increases in diving effort have been observed in several species with effort increasing to either support the growing needs of the young [32], [56] or adapt to seasonal fluctuations in prey available [28], [84]. In the present study, seasonal differences were found in all of the foraging effort indices measured.

Foraging effort in the current study increased as the pup provisioning period progressed. This was seen as an increase in the proportion of time spent diving within a foraging trip, which was the product of an increase in mean dive duration and dive rate and a decrease in the mean post-dive duration. The proportion of time spent diving peaked around the middle of August as a result of a peak in mean dive duration and vertical travel rate. Arnould and Hindell [36] determined that peak milk delivery to pups occurred around mid-July at 7.5 months of age, reducing gradually until weaning in early October. These results suggest that, shortly after the peak nutritional demands of lactation, mothers reach a plateau in the time they can spend diving. However mean post-dive and dive duration continue to decrease beyond this point, suggesting females are encountering further energetic limitations. Like all otariids, female AUFS become pregnant within a few days of giving birth to their current pup, they then undergo a three-month diapause and by mid-August are entering the third trimester of pregnancy [37], [38]. The observed reduction in mean dive duration beyond mid-August may reflect the physiological limits placed on the female by the increasing metabolic demands (e.g. oxygen) of the rapidly growing foetus [85].

Foraging effort as a function of the whole foraging cycle was found to, generally, increase as the pup provisioning period progressed. This was achieved by increasing the proportion of time spent diving within foraging trips and between May–August spending less time ashore than other months. However, individuals did not show any change in the foraging trip duration between months. Individuals are limited in the time that can be spent foraging by the fasting abilities of their waiting young, as such, their ability to increase their overall foraging trip duration without having a negative impact on the growth and development of their young may be limited [86].

Concurrent with females having longer dive durations, increasing vertical travel rate and proportion of time spent diving in winter, individuals spent significantly less time ashore between May–August than in other months. This could reflect a strategy by females of reducing their time on land to maximise potential foraging time at sea during the period when the nutritional demands of lactation are greatest. As foraging effort (proportion of time diving) does not decrease thereafter, the longer periods ashore in September may be due to females extending their periods ashore while waiting to be reunited with their young which are spending increase periods of times away from the colony at this time [87]. Increasing periods ashore in response to delayed reunion with pups has been observed in northern fur seals [88].

Foraging effort in species is inherently linked to the spatial distribution of forage items and the horizontal movement patterns of the foraging individual [89], [90]. This study's reliance on TDR data meant that is was not able to assess the spatial patterns of foraging effort for this species. As such, this study is limited in its' ability to provide conclusions on the links between spatial movements and the temporal structure of foraging effort. However, the results of the current study highlight the complex temporal pattern of foraging effort used by female AUFS. The factors limiting foraging effort (SDA or prey availability) appeared to operate both within a single day and across a foraging trip. Furthermore, the present study also displayed the response by females to the increasing demands of their growing young. Future studies linking the temporal changes in foraging effort with feeding success and spatial movement patterns will provide for a greater understanding of the linkages between these temporal shifts in activity and the acquisition of resources in Australian fur seals.

## Supporting Information

Table S1 Summary deployment information and dive behaviour for female Australian fur seals (*Arctocephalus pusillus doriferus*) provision pups at Kanowna Island, northern Bass Strait, Australia.
(DOCX)
