# Supplementary material for: Temporal Allocation of Foraging Effort in Female Australian Fur Seals (Arctocephalus pusillus doriferus)
Source: PLoS One. 2013 Nov 14;8(11):e79484. doi: 10.1371/journal.pone.0079484 (PMC3828376; doi:10.1371/journal.pone.0079484)
Supplement: Table S1 — Summary deployment information and dive behaviour for female Australian fur seals (Arctocephalus pusillus doriferus) provision pups at Kanowna Island, northern Bass Strait, Australia. (DOCX) [file pone.0079484.s001.docx]

Table S1: Summary deployment information and dive behaviour for female Australian fur seals (*Arctocephalus pusillus doriferus*) provision pups at Kanowna Island, northern Bass Strait, Australia.

| **ID** | **Year** | **Month** | **Deployment dur (d)** | **No. of trips** | **Mean trip dur (d)** | **Mean prop time diving p/trip** | **Mean dive time (s)** | **Mean dives p/day** | **Prop time diving p/day** |
| --- | --- | --- | --- | --- | --- | --- | --- | --- | --- |
| 1 | 1998 | Feb | 123.9 | 19 | 4.7 ± 1.8 | 0.35 ± 0.1 | 163.9 ± 55.6 | 170.6 ± 72.5 | 0.36 |
| 2 | 1998 | Apr | 59 | 6 | 7.8 ± 2.8 | 0.38 ± 0.02 | 160.3 ± 49.4 | 168.5 ± 53.1 | 0.38 |
| 3 | 1998 | Apr | 5.8 | 2 | 2.3 ± 2.7 | 0.41 ± 0.04 | 140.6 ± 54.7 | 195.3 ± 134.5 | 0.44 |
| 4 | 1998 | Jul | 3.7 | 1 | 3.7 | 0.43 | 212.6 ± 60.8 | 139.9 ± 65.9 | 0.42 |
| 5 | 1998 | Jul | 6.6 | 2 | 3.3 ± 2.4 | 0.47 ± 0.04 | 265.2 ± 72.9 | 159.1 ± 57.9 | 0.49 |
| 6 | 1998 | Jul | 8.3 | 4 | 1.6 ± 1 | 0.53 ± 0.08 | 210.5 ± 76.1 | 147.8 ± 81.8 | 0.52 |
| 7 | 1998 | Jul | 125.4 | 18 | 4.9 ± 1.9 | 0.43 ± 0.06 | 191.4 ± 47.1 | 188.8 ± 59.1 | 0.43 |
| 8 | 1999 | Mar | 69.3 | 14 | 3.4 ± 1.7 | 0.29 ± 0.08 | 196.2 ± 71 | 117 ± 64.9 | 0.31 |
| 9 | 1999 | Mar | 69.4 | 10 | 5 ± 1.8 | 0.32 ± 0.08 | 162.9 ± 70.4 | 159.6 ± 72.7 | 0.33 |
| 10 | 1999 | May | 65.6 | 22 | 2.1 ± 1.5 | 0.5 ± 0.11 | 129.4 ± 51.4 | 295.1 ± 141 | 0.49 |
| 11 | 2002 | May | 65.1 | 14 | 3.3 ± 2.2 | 0.4 ± 0.09 | 249 ± 68.9 | 136.9 ± 59.7 | 0.39 |
| 12 | 2002 | May | 65.7 | 9 | 5.2 ± 2.4 | 0.47 ± 0.06 | 252.5 ± 72 | 159.8 ± 45.9 | 0.47 |
| 13 | 2003 | Jun | 59 | 41 | 0.8 ± 0.4 | 0.47 ± 0.07 | 169.9 ± 35.2 | 223.2 ± 127.7 | 0.47 |
| 14 | 2003 | Jun | 88.4 | 38 | 1.4 ± 1 | 0.51 ± 0.08 | 86.9 ± 60.4 | 367.7 ± 138.4 | 0.51 |
| 15 | 2003 | Jun | 65.4 | 9 | 5.9 ± 1.4 | 0.4 ± 0.05 | 215.3 ± 65.8 | 139.4 ± 48.4 | 0.4 |
| 16 | 2003 | Aug | 7.5 | 2 | 2.8 ± 0.3 | 0.38 ± 0.08 | 235.1 ± 87 | 135.9 ± 67.2 | 0.37 |
| 17 | 2003 | Aug | 6.7 | 2 | 2.3 ± 0.7 | 0.41 ± 0.02 | 201.4 ± 70.2 | 147.8 ± 46.9 | 0.42 |
| 18 | 2003 | Aug | 8.5 | 4 | 1.1 ± 0.5 | 0.37 ± 0.06 | 147.5 ± 90.7 | 125.5 ± 58.5 | 0.36 |
| 19 | 2004 | Jul | 4.8 | 1 | 4.8 | 0.31 | 211.5 ± 51 | 112.9 ± 74.1 | 0.31 |
| 20 | 2004 | Jul | 10.7 | 2 | 4.9 ± 1.3 | 0.34 ± 0 | 81.2 ± 97.5 | 151.1 ± 54.1 | 0.33 |
| 21 | 2004 | Jul | 14.7 | 2 | 6.1 ± 0.3 | 0.35 ± 0 | 202.8 ± 94.7 | 175.9 ± 56.3 | 0.35 |
| 22 | 2005 | Jun | 16.5 | 7 | 1.6 ± 1.1 | 0.41 ± 0.04 | 126.1 ± 55.5 | 188.3 ± 69.4 | 0.45 |
| 23 | 2005 | Jun | 43.4 | 18 | 1.4 ± 0.7 | 0.39 ± 0.11 | 159.2 ± 67.3 | 162.7 ± 74.6 | 0.41 |
| 24 | 2005 | Jun | 48.3 | 12 | 3 ± 2 | 0.29 ± 0.1 | 173.4 ± 115.5 | 105.9 ± 56.3 | 0.26 |
| 25 | 2005 | Jun | 41.1 | 7 | 4.4 ± 1.5 | 0.39 ± 0.08 | 220.2 ± 57.2 | 144.5 ± 56.1 | 0.37 |
| 26 | 2005 | Jun | 99.7 | 24 | 2.8 ± 2.4 | 0.37 ± 0.09 | 171 ± 72.3 | 186.7 ± 99.7 | 0.36 |
| 27 | 2006 | Jun | 3.5 | 1 | 3.5 | 0.24 | 293.8 ± 113.2 | 92.8 ± 64 | 0.28 |
| 28 | 2006 | Jun | 44.2 | 8 | 4.1 ± 1.2 | 0.37 ± 0.05 | 161.7 ± 40.6 | 177.7 ± 58 | 0.37 |
| 29 | 2006 | Jun | 4 | 1 | 4 | 0.40 | 209.2 ± 93.1 | 153.6 ± 60.2 | 0.4 |
| 30 | 2006 | Jun | 40.9 | 5 | 6.6 ± 1.3 | 0.32 | 169 ± 52.8 | 151.7 ± 44.6 | 0.33 |
| 31 | 2006 | Jun | 1.9 | 1 | 1.9 | 0.37 | 27.2 ± 97.7 | 241.7 ± 151.9 | 0.38 |
| 32 | 2007 | Apr | 59.6 | 26 | 1.1 ± 0.8 | 0.43 ± 0.13 | 207.4 ± 68.5 | 138.5 ± 82.6 | 0.4 |
| 33 | 2007 | Apr | 8.6 | 3 | 1.6 ± 1 | 0.32 ± 0.11 | 184.9 ± 74.4 | 144.9 ± 88.5 | 0.36 |
| 34 | 2007 | Apr | 49.7 | 8 | 4.4 ± 0.8 | 0.44 ± 0.06 | 210.4 ± 62.7 | 166.3 ± 78.3 | 0.45 |
| 35 | 2007 | Apr | 47.1 | 17 | 1.9 ± 1.9 | 0.41 ± 0.12 | 161.7 ± 62.7 | 172.4 ± 94.7 | 0.38 |
| 36 | 2007 | Apr | 62.2 | 15 | 2.8 ± 1.4 | 0.4 ± 0.08 | 191.4 ± 81 | 173.8 ± 76.5 | 0.41 |
| 37 | 2007 | Jul | 3.7 | 1 | 3.7 | 0.43 | 282.1 ± 66.8 | 121.7 ± 61.9 | 0.43 |
| 38 | 2007 | Jul | 4.7 | 2 | 2.3 ± 2.2 | 0.59 ± 0.11 | 237.7 ± 59.7 | 191.6 ± 50.8 | 0.53 |
| 39 | 2008 | May | 9.1 | 2 | 3.4 ± 1.5 | 0.45 ± 0.07 | 251.3 ± 79.6 | 157 ± 48.7 | 0.43 |
| 40 | 2008 | May | 6.2 | 4 | 1.2 ± 1.4 | 0.39 ± 0.09 | 182.2 ± 76.9 | 171.1 ± 132 | 0.41 |
| 41 | 2008 | May | 21.7 | 12 | 1.3 ± 1.6 | 0.47 ± 0.13 | 104.7 ± 74.9 | 270.4 ± 151 | 0.39 |
| 42 | 2008 | May | 2.5 | 1 | 2.5 | 0.40 | 240.4 ± 66.2 | 118.3 ± 58.8 | 0.4 |
| 43 | 2008 | May | 3.8 | 1 | 3.8 | 0.33 | 248.4 ± 80.8 | 120.4 ± 85.6 | 0.33 |
| 44 | 2008 | May | 9.4 | 3 | 1.8 ± 1.2 | 0.46 ± 0.04 | 284.5 ± 82.8 | 144.2 ± 73.5 | 0.49 |
| 45 | 2008 | May | 14.2 | 2 | 5.9 ± 0.6 | 0.39 ± 0.01 | 232.6 ± 75.2 | 139.2 ± 37.8 | 0.4 |
| 46 | 2008 | Jun | 41.5 | 19 | 1.4 ± 1.2 | 0.47 ± 0.09 | 226.1 ± 60.3 | 156.5 ± 81.9 | 0.42 |
| 47 | 2008 | Jun | 42.9 | 9 | 3.5 ± 1.8 | 0.47 ± 0.06 | 257.4 ± 68.4 | 161.1 ± 45.3 | 0.45 |
| 48 | 2008 | Jul | 4.7 | 5 | 0.5 ± 0.1 | 0.70 ± 0.06 | 194.7 ± 37.3 | 247.7 ± | 0.67 |
| 49 | 2008 | Jul | 4.1 | 1 | 4.1 | 0.30 | 161.2 ± 43.6 | 163.7 ± 26 | 0.32 |
| 50 | 2008 | Jul | 4.1 | 1 | 4.1 | 0.37 | 171.6 ± 47.6 | 153.4 ± 49.6 | 0.38 |
| 51 | 2009 | May | 9.8 | 4 | 1.4 ± 0.8 | 0.47 ± 0.09 | 212.5 ± 60 | 169.5 ± 78.9 | 0.44 |
| 52 | 2009 | May | 9.9 | 3 | 3 ± 3 | 0.42 ± 0.09 | 201.5 ± 61.9 | 153.1 ± 63.8 | 0.42 |
| 53 | 2009 | May | 7 | 3 | 1.7 ± 1.1 | 0.53 ± 0.12 | 205.3 ± 65.3 | 206.7 ± 69.9 | 0.5 |
| 54 | 2009 | May | 16.9 | 7 | 1.7 ± 1.1 | 0.43 ± 0.05 | 138.1 ± 45.6 | 217.4 ± 121.4 | 0.41 |
| 55 | 2009 | May | 35 | 6 | 4.9 ± 3.8 | 0.35 ± 0.08 | 159.9 ± 73.7 | 168.3 ± 71.3 | 0.31 |
| 56 | 2009 | May | 38.8 | 6 | 4.8 ± 1.7 | 0.41 ± 0.07 | 160.6 ± 44.5 | 210.9 ± 64.4 | 0.4 |
| 57 | 2009 | May | 3.4 | 1 | 3.4 | 0.32 | 205.5 ± 80.3 | 138.3 ± 78.1 | 0.33 |
| 58 | 2009 | Jun | 3.7 | 1 | 3.7 | 0.47 | 246.2 ± 64 | 172.3 ± 97 | 0.47 |
| 59 | 2009 | Jun | 3.8 | 2 | 1.8 ± 2 | 0.19 | 115.6 ± 68.5 | 107.9 ± 55.4 | 0.33 |
| 60 | 2009 | Jun | 7.5 | 1 | 7.5 | 0.33 | 243 ± 55.2 | 124.6 ± 49.8 | 0.34 |
| 61 | 2009 | Jun | 4.3 | 1 | 4.3 | 0.36 | 201.5 ± 54.7 | 141 ± 52 | 0.37 |
| 62 | 2009 | Jun | 5.5 | 1 | 5.5 | 0.40 | 181.8 ± 28.7 | 176.3 ± 77.9 | 0.42 |
| 63 | 2009 | Jun | 9.6 | 1 | 9.6 | 0.36 | 142.8 ± 68.8 | 212.4 ± 87.7 | 0.36 |
| 64 | 2009 | Jun | 27.8 | 17 | 1.1 ± 0.8 | 0.43 ± 0.10 | 174.4 ± 56.8 | 200.4 ± 144.5 | 0.45 |
| 65 | 2009 | Jun | 10.6 | 6 | 1.3 ± 1 | 0.49 ± 0.14 | 206.7 ± 69.4 | 204 ± 97.4 | 0.48 |
| 66 | 2009 | Jun | 8.7 | 3 | 2.1 ± 2.3 | 0.50 ± 0.050 | 196.3 ± 59.4 | 187.8 ± 86.2 | 0.51 |
| 67 | 2009 | Jun | 23.9 | 4 | 4.7 ± 2.5 | 0.44 ± 0.11 | 264.7 ± 89.4 | 146 ± 59.4 | 0.45 |
| 68 | 2009 | Jul | 138.2 | 19 | 5.34 ± 3.6 | 0.49 ± 0.10 | 177.5 ± 49.1 | 264 ± 0.9 | 0.44 |
| **Sample means** | | | **31.5 ± 35.0** | **8.2 ± 8.8** | **2.9 ± 2.4** | **0.41 ± 0.07** | **186.9 ± 54.4** | **201.5 ± 62.5** | **0.40 ± 0.01** |
